# Difference in health status of Korean farmers according to gender

**DOI:** 10.1186/s40557-019-0287-7

**Published:** 2019-03-08

**Authors:** Ho Lee, Seong-yong Cho, Jin-seok Kim, Seong-yong Yoon, Bu-il Kim, Jong-min An, Ki-beom Kim

**Affiliations:** Department of Occupational and Environmental Medicine, Soonchunhyang University Gumi Hospital, 179, 1gongdan-ro, Gumi, Gyeongsangbuk-do Republic of Korea

**Keywords:** Farmer, Gender, Health status, Lifestyle diseases, Musculoskeletal pain, Psychosocial stress, Self-health awareness

## Abstract

**Background:**

The objective of this study was to compare differences in lifestyle diseases, musculoskeletal pain, psychosocial stress, and self-health awareness according to gender in Korean farmers.

**Methods:**

The study population comprised 436 farmers residing in rural areas in Korea. A self-administered questionnaire was used to survey demographic characteristics, health-related behaviors, and musculoskeletal pain. The psychosocial well-being index short form (PWI-SF) was used to survey psychosocial stress, and the 12-item short form health survey (SF-12) was used to survey self-health awareness. In addition, a clinical examination was performed for each participant, and lifestyle diseases were identified through a health checkup.

**Results:**

Among lifestyle diseases, females showed a significantly higher proportion than males for metabolic syndrome (OR: 4.57 [95% CI, 1.67–12.51]). For musculoskeletal pain, females again showed significantly higher proportion than males for hand pain (OR: 16.79 [95% CI, 3.09–91.30]), and pain in at least one body part (OR: 2.34 [95% CI, 1.16–4.70]). For psychosocial stress, females showed a significantly higher proportion than males for high-risk stress (OR: 3.10 [95% CI, 1.17–8.24]). Among the items in self-health awareness, females showed significantly higher proportion than males for mental component score (MCS) (OR: 3.10 [95% CI, 1.52–6.31]) and total score (OR: 2.34 [95% CI, 1.11–4.90]).

**Conclusions:**

For all items that showed significant differences, females showed higher proportion than males, which indicates that female farmers tended to have poorer overall health than male farmers. Therefore, specialized programs will have to be developed to improve the health of female farmers.

## Background

The rural population of Korea has declined sharply, from 10.8 million in 1980 to 2.4 million in 2017. During this time, young people from rural areas had relocated to urban areas, creating an aging society in rural regions. This phenomenon has created a shortage of labor in younger age groups, while increasing the intensity of labor for elderly and female farmers [1, 2].

Farming, which is known to be a dangerous occupation for both males and females, has unique characteristics that are different from other occupations due to the characteristics and behaviors of farmers, their working environment, and organizational structure [3]. Moreover, farmers do not properly apply the safety rules, and their financial situation is also unstable [4]. In Korea, occupational injuries within the farming sector have higher-than-average accident rates reported than other occupations [5, 6]. In addition, the basic living conditions of Korean farmers are much poorer than those living in urban areas due to excessive physical labor, increase in the number of female farmers, lack of education, poor hygienic environment, apathy towards health, and low socioeconomic status. They also experience difficulties in the utilization of healthcare facilities. Furthermore, they must also participate in other outdoor and household work due to a shortage of labor in farming areas. The physical and mental functions of farmers tend to deteriorate as a consequence [7–9].

A study in 2009 compared the proportion of musculoskeletal and chronic diseases between Korean farmers and other occupational groups; it found that both male and female farmers showed a higher proportion of musculoskeletal disease, while female farmers showed a significantly higher proportion of hypertension than other occupational groups [10]. In a study conducted in 2016 on the proportion of musculoskeletal pain and the characteristics of Korean farmers, female farmers showed a significantly higher risk of pain in the shoulders, hands, lower back, and legs compared to male farmers [11]. A study in 2015 examined the health status and related factors of farmers, using the 12-item short form health survey (SF-12) to evaluate self-health awareness; the results showed that females had a lower mental component score (MCS) than males [12].

As shown, studies have compared differences in the risk or proportion of specific diseases between male and female farmers or differences in disease proportion between farmers and other occupational groups. However, there have been no studies that systematically compare the physical and mental state of farmers according to gender. Accordingly, this study aimed to compare differences in lifestyle diseases, musculoskeletal pain, psychosocial stress, and self-health awareness of Korean farmers according to gender.

## Methods

### Subjects

The study area was set as rural areas in Gyeongsangbuk-do Province in Korea. The study population consisted of farmers residing in a total of 11 areas: 3 areas in 2015, 4 in 2016, and 4 in 2017. Among the 458 people who participated in both a questionnaire survey and health checkup conducted by the National Health Insurance Service (NHIS), 436 people were included in the final study population, after excluding 22 people who did not work in farming or provided incomplete responses to the questionnaire.

### Survey content

A self-administered questionnaire was used to survey demographic characteristics, health-related behaviors, and musculoskeletal pain. The specific details were as follows:

#### Demographic characteristics, health-related behaviors, and clinical examination

The demographic characteristics of the subjects included: gender, age, working duration, main crops, presence of family members other than the spouse, spouse, income, and housework time. Main crops were categorized as grains, vegetables, fruits, livestock, and other. Spouse was categorized as “Yes” or “No” (single, divorced, or widowed), and presence of family members other than the spouse was categorized as “Yes” or “No”. Income was categorized as < 10 million won, 10–24 million won, 25–49 million won, and ≥ 50 million won, and housework was categorized as 0, < 2, and ≥ 2 h/day.

Alcohol drinking, smoking, and exercise status were surveyed as health-related behaviors. Alcohol drinking status was categorized as nondrinker, once/week, and two or more times/week. Smoking status was categorized as nonsmoker, ex-smoker, and current smoker. Exercise status was categorized as “Yes,” if the subjects performed moderate to vigorous exercise or walking at least 5 days a week and “No,” if otherwise.

A clinical examination was performed on each participant through a health checkup to measure height, weight, waist circumference, body mass index (BMI), blood pressure, hemoglobin, fasting blood glucose (FBS), serum lipids, and serum liver enzymes. Obesity was defined based using BMI, with BMI < 25 kg/m^2^ as normal and ≥ 25 kg/m^2^ as obese [13]. Blood pressure, hemoglobin, FBS, serum lipids, and serum liver enzymes were defined as abnormal when a disease was suspected or diagnosed based on the standards of the NHIS in Korea. The details are as follows. Hypertension was defined as systolic pressure ≥ 140 mmHg or diastolic pressure ≥ 90 mmHg during blood pressure measurement, or being treated for hypertension. Diabetes mellitus was defined as FBS ≥126 mg/dL, or being treated for diabetes mellitus. Dyslipidemia was defined as total cholesterol ≥240 mg/dL, triglyceride ≥200 mg/dL, high-density lipoprotein cholesterol (HDL-C) < 40 mg/dL, low-density lipoprotein cholesterol (LDL-C) ≥160 mg/dL, or being treated for dyslipidemia. Anemia was defined as hemoglobin < 13 mg/dL for males and < 12 mg/dL for females. For serum liver enzymes, aspartate aminotransferase (AST), alanine aminotransferase (ALT), and gamma-glutamyltransferase (γ-GTP) levels were measured, and AST ≥50 IU/L, ALT ≥45 IU/L, or γ-GTP ≥78 IU/L for males and ≥ 46 IU/L for females were considered abnormal [14].

For metabolic syndrome, the National Cholesterol Education Program’s Adult Treatment Panel III (NCEP ATP III) was applied for metabolic syndrome, along with the International Diabetes Federation (IDF) definition in 2009 for waist circumference. Those who satisfied 3 or more of the following conditions were considered to have metabolic syndrome: systolic blood pressure ≥ 130 mmHg, diastolic blood pressure ≥ 85 mmHg, or being treated for hypertension; FBS ≥100 mg/dL or being treated for diabetes mellitus; waist circumference ≥ 90 cm for males and ≥ 80 cm for females; triglyceride ≥150 mg/dL; and HDL-C < 40 mg/dL for males and < 50 mg/dL for females [15–17].

Lifestyle diseases were identified based on these results. Lifestyle diseases refer to a disease group with onset and progression affected by lifestyle, including diet, exercise, smoking, and drinking [18]. In this study, lifestyle disease was defined as a suspected or confirmed disease in the health checkup or diagnosis with metabolic syndrome. Specifically, hypertension, diabetes mellitus, dyslipidemia, anemia, abnormal serum liver enzymes, and metabolic syndrome were checked as lifestyle diseases.

#### Musculoskeletal pain assessment

To evaluate musculoskeletal pain symptoms, this study used the questionnaire “Guidelines for surveys of harmful factors in tasks involving musculoskeletal loads” from the Korea Occupational Safety and Health Agency (KOSHA) CODE H-9-2016 [19]. Items included in the questionnaire were: six specific body parts (neck, shoulder, arm, hand, lower back, and leg), duration of pain, severity of pain, and frequency of symptoms in the last year. Based on the results, musculoskeletal pain was defined as moderate-to-severe pain in one or more areas that persists for at least one week or occurs more than once in a month, in accordance with Standard 2 of the National Institute for Occupational Safety and Health (NIOSH) [20].

#### Psychosocial stress assessment

The psychosocial well-being index short form (PWI-SF) was used as the tool for assessing psychosocial stress. The form comprised questions about physical and mental state in the past few weeks, with the total score ranging between 0 and 54 points. Higher scores indicated a higher level of psychosocial stress, with ≤8, 9–26, and ≥ 27 points defined as healthy, potential stress, and high-risk stress, respectively [21, 22].

#### Self-health awareness assessment

The 12-item short form health survey (SF-12) was used as the tool for assessing self-health awareness. SF-12 is an abridged version of SF-36, which can be used to measure physical component score (PCS) and sub-items, mental component score (MCS) and sub-items, and the total score. Sub-items under PCS included physical functioning (PF), role physical (RP), bodily pain (BP), and general health (GH); sub-items under MCS included mental health (MH), role emotional (RE), social functioning (SF), and vitality (VT). A higher score in each item indicated better-perceived health status for that item [23–25].

### Statistical analysis

In this study, t-test and chi-square test were performed to investigate the differences in demographic characteristics, health-related behaviors, clinical examination, musculoskeletal pain, and self-health awareness between male and female farmers. A linear-by-linear association test was performed to investigate differences in psychosocial stress. In addition, multiple logistic regression analysis was performed to investigate the differences in lifestyle disease, musculoskeletal pain, psychosocial stress, and self-health awareness between male and female farmers. For psychosocial stress, healthy and potential stress of PWI-SF was set as low risk and used as the reference. For self-health awareness, the results were divided into high and low based on the median value of SF-12 scores, with the higher score group set as the reference. The adjustment variables included in multiple logistic regression analysis were age, spouse, income, housework time, alcohol drinking, smoking, exercise; they were included in the analysis because showed *p*-value < 0.15 in univariate analysis. We also included several other variables (i.e., work duration, main crops, presence of family members other than the spouse) associated with lifestyle disease, musculoskeletal pain, psychosocial stress, and self-health awareness in previous study [26–30]. All statistical analyses were performed using SPSS version 14.0 (SPSS, Inc., Chicago, IL, USA).

## Results

Among demographic characteristics, the mean ages of males and females were 62.7 ± 9.21 and 60.9 ± 9.67 years, respectively. The proportion of males and females without a spouse was 8.8 and 19.0%, respectively. The proportion of males and females who did no housework was 49.0 and 1.3%, respectively, while 37.3% of males and 36.6% of females spent < 2 h/day on housework, and 13.7% of males and 62.1% of females spent ≥2 h/per day on housework. There were no differences in working duration, main crops, presence of family members other than the spouse, and income between males and females.

Among health-related behaviors, the proportion of male and female nondrinkers was 38.2 and 81.0%, respectively, while 16.7% of males and 12.9% of females drank once a week, and 45.1% of males and 6.0% of females drank two or more times a week. The proportion of male and female nonsmokers was 36.3 and 97.4%, respectively, while 31.9% of males and 1.7% of females were ex-smokers, and 31.9% of males and 0.9% of females were current smokers. There was no difference in exercise level between males and females (*p* < 0.05) (Table 1).

**Table 1:** Baseline characteristics of the study subjects according to gender

Variables	Male	Female	*p*-value^*^
	n (%) or Mean ± SD	n (%) or Mean ± SD	
Demographic variables			
Age (years)^a^	62.7 ± 9.21	60.9 ± 9.67	0.048
Work duration (years)^a^	29.2 ± 18.5	29.1 ± 16.8	0.950
Main crops^b^			0.952
Grains	34 (16.7)	42 (18.1)	
Vegetables	54 (26.5)	65 (28.0)	
Fruits	106 (52.0)	115 (49.6)	
Livestock	8 (3.9)	7 (3.0)	
Other	2 (1.0)	3 (1.3)	
Spouse^b^			0.002
No	18 (8.8)	44 (19.0)	
Yes	186 (91.2)	187 (81.0)	
Presence of family members other than the spouse^b^			0.903
No	66 (32.4)	76 (32.9)	
Yes	138 (67.6)	155 (67.1)	
Income (million KRW)^b^			0.133
< 10	60 (32.8)	89 (41.8)	
10–24	38 (20.8)	47 (22.1)	
25–49	64 (35.0)	53 (24.9)	
≥ 50	21 (11.5)	24 (11.3)	
Housework time (hours/day)^b^			0.000
0	100 (49.0)	3 (1.3)	
< 2	76 (37.3)	85 (36.6)	
≥ 2	28 (13.7)	144 (62.1)	
Health-related behaviors^b^			
Alcohol drinking			0.000
Nondrinker	78 (38.2)	188 (81.0)	
1/week	34 (16.7)	30 (12.9)	
More than 2/week	92 (45.1)	14 (6.0)	
Smoking			0.000
Nonsmoker	74 (36.3)	225 (97.4)	
Ex-smoker	65 (31.9)	4 (1.7)	
Current smoker	65 (31.9)	2 (0.9)	
Exercise			0.124
No	110 (53.9)	142 (61.2)	
Yes	94 (46.1)	90 (38.8)	

KRW South Korean won.

*p-value by t-test or Chi-square test

^a^Mean ± SD

^b^N (%)

When comparing lifestyle diseases between males and females, proportion of diabetes mellitus was significantly lower in females (9.1%) than in males (17.2%); anemia was significantly higher in females (15.5%) than in males (6.4%); abnormal serum liver enzymes were significantly lower in females (7.8%) than in males (23.5%); and metabolic syndrome was significantly higher in females (32.6%) than in males (21.6%). Meanwhile, there were no differences in hypertension, dyslipidemia, and obesity between males and females (*p* < 0.05) (Table 2).

**Table 2:** Comparison of lifestyle diseases according to gender

Variables	Male	Female	*p*-value^*^
	n (%)	n (%)	
Hypertension			0.843
No	125 (61.3)	140 (60.3)	
Yes	79 (38.7)	92 (39.7)	
Diabetes mellitus			0.012
No	169 (82.8)	211 (90.9)	
Yes	35 (17.2)	21 (9.1)	
Dyslipidemia			0.952
No	119 (58.3)	136 (58.6)	
Yes	85 (41.7)	96 (41.4)	
Anemia			0.003
No	191 (93.6)	196 (84.5)	
Yes	13 (6.37)	36 (15.5)	
Abnormal serum liver enzymes			0.001
No	156 (76.5)	206 (88.8)	
Yes	48 (23.5)	26 (11.2)	
Obesity			0.703
No	125 (61.3)	138 (59.5)	
Yes	79 (38.7)	94 (40.5)	
Metabolic syndrome			0.025
No	120 (78.4)	120 (67.4)	
Yes	33 (21.6)	58 (32.6)	

*p-value by Chi-square test

When comparing the complaint rate of males and females experiencing musculoskeletal pain, 5.4% of males and 12.1% of females had neck pain; 4.0% of males and 19.0% of females had hand pain; 24.8% of males and 40.1% of females had lower back pain; and 25.7% of males and 37.9% of females had leg pain. These results show a significantly higher proportion of females having neck, hand, lower back, and leg pain. Moreover, the proportion of those with pain in at least one body part was significantly higher in females (67.2%) than in males (47.0%). Meanwhile, there were no differences in the shoulder and arm pain between males and females (*p* < 0.05) (Table 3).

**Table 3:** Comparison of musculoskeletal pain according to body parts according to gender

Variables	Male	Female	*p*-value^*^
	*n* = 202 (46.5%)	*n* = 232 (53.5%)	
Neck			0.016
No	191 (94.6)	204 (87.9)	
Yes	11 (5.4)	28 (12.1)	
Shoulder			0.066
No	160 (79.2)	166 (71.6)	
Yes	42 (20.8)	66 (28.4)	
Arm			0.397
No	178 (88.1)	198 (85.3)	
Yes	24 (11.9)	34 (14.7)	
Hand			0.000
No	194 (96.0)	188 (81.0)	
Yes	8 (4.0)	44 (19.0)	
Back			0.001
No	152 (75.2)	139 (59.9)	
Yes	50 (24.8)	93 (40.1)	
Leg			0.007
No	150 (74.3)	144 (62.1)	
Yes	52 (25.7)	88 (37.9)	
Pain in at least one body part			0.000
No	107 (53.0)	76 (32.8)	
Yes	95 (47.0)	156 (67.2)	

*p-value by Chi-square test

When comparing psychosocial stress between males and females using the PWI-SF, 27.1% of males and 16.7% of females belonged to the healthy group; 60.3% of males and 58.1% of females belonged to the potential stress group; and 12.6% of males and 25.2% of females belonged to the high-risk stress group (*p* < 0.05) (Table 4).

**Table 4:** Comparison of psychosocial stress according to gender

Variables	Male	Female	*p*-value^*^
	n (%)	n (%)	
Group			0.000
Healthy	54 (27.1)	37 (16.7)	
Potential stress	120 (60.3)	129 (58.1)	
High-risk stress	25 (12.6)	56 (25.2)	

*p-value of linear-by-linear association

When comparing self-health awareness between males and females using the SF-12, PCS was 68.6 ± 23.5 in males and 58.3 ± 26.0 in females. MCS was 77.5 ± 18.8 in males and 67.8 ± 22.5 in females. The total score was 73.1 ± 18.9 in males and 63.1 ± 22.6 in females. Females showed significantly lower PCS, MCS, individual sub-item (PF, RP, BP, GH, MH, RE, SF, or VT) scores, and total score in the SF-12, compared to males (*p* < 0.05) (Table 5).

**Table 5:** Comparison of self-health awareness according to gender

Variables	Male	Female	*p*-value^*^
	Mean ± SD	Mean ± SD	
PCS	68.6 ± 23.5	58.3 ± 26.0	0.000
PF	71.8 ± 33.7	60.2 ± 35.8	0.001
RP	75.3 ± 31.5	63.8 ± 35.4	0.000
BP	81.1 ± 24.5	70.8 ± 30.5	0.000
GH	46.4 ± 25.0	38.4 ± 24.7	0.001
MCS	77.5 ± 18.8	67.8 ± 22.5	0.000
MH	76.1 ± 23.3	66.5 ± 26.1	0.000
RE	82.6 ± 26.6	73.2 ± 29.8	0.001
SF	89.4 ± 22.9	80.7 ± 29.3	0.001
VT	62.1 ± 33.2	50.8 ± 34.2	0.001
Total score	73.1 ± 18.9	63.1 ± 22.6	0.000

PCS physical component score, PF physical functioning, RP role physical, BP bodily pain, GH general health, MCS mental component score, MH mental health, RE role emotional, SF social functioning, VT vitality.

*p-value by t-test

Multiple logistic regression analysis was performed to investigate the differences in lifestyle diseases, musculoskeletal pain, psychosocial stress, and self-health awareness between males and females, after adjusting for demographic characteristics and health-related behaviors. Among lifestyle diseases, the risk for metabolic syndrome was significantly higher in females than males (OR: 4.57 [95% CI, 1.67–12.51]). For musculoskeletal pain, females showed significantly higher risk than males for hand pain (OR: 16.79 [95% CI, 3.09–91.30]), and pain in at least one body part (OR: 2.34 [95% CI, 1.16–4.70]). For psychosocial stress, females had a significantly higher risk for high-risk stress than males (OR: 3.10 [95% CI, 1.17–8.24]). Among the items in self-health awareness, females showed significantly higher risk than males for MCS (OR: 3.10 [95% CI, 1.52–6.31]) and total score (OR: 2.34 [95% CI, 1.11–4.90]) (Table 6).

**Table 6:** Adjusted odds ratio of lifestyle diseases, musculoskeletal pain, psychosocial stress, and self-health awareness according to gender

Variables	Female	95% CI
	OR	
Lifestyle disease		
Hypertension	1.42	0.70–2.87
Diabetes mellitus	0.53	0.21–1.36
Dyslipidemia	1.24	0.62–2.45
Anemia	1.54	0.50–4.71
Abnormal serum liver enzymes	1.40	0.52–3.75
Obesity	2.05	1.02–4.13
Metabolic syndrome	4.57	1.67–12.51
Musculoskeletal pain		
Neck	0.90	0.25–3.28
Shoulder	0.75	0.34–1.65
Arm	0.67	0.25–1.80
Hand	16.79	3.09–91.30
Lower back	2.12	0.99–4.56
Leg	1.42	0.67–3.03
Pain in at least one body part	2.34	1.16–4.70
Psychosocial stress		
High-risk stress	3.10	1.17–8.24
Self-health awareness		
PCS	1.52	0.75–3.11
MCS	3.10	1.52–6.31
Total score	2.34	1.11–4.90

PCS physical component score, MCS mental component score.

Adjusted for age, working duration, main crops, presence of family members other than the spouse, spouse, income, housework time, alcohol drinking, smoking, exercise.

OR of male: 1.00.

Odds ratio and 95% confidence interval by multiple logistic regression analysis.

## Discussion

In this study, the proportion of metabolic syndrome was significantly higher in females (32.6%) than in males (21.6%), and the risk of metabolic syndrome in females was 4.57 [95% CI, 1.67–12.51] times higher than in males. In a study that followed up 1095 rural residents for 5 years to measure the proportion of metabolic syndrome, females showed a significantly higher proportion of 46.4/1000 person-years, compared to 30.0/1000 person-years for males, which is consistent with the present study [31]. A previous study of 91 farmers found that the proportion of metabolic syndrome was lower in females (42.9%) than in males (51.4%), which is contradictory to the present study [32]. The previous study did not include people being treated for hypertension and diabetes mellitus in the criteria for metabolic syndrome. This is postulated to be the reason for the difference from the present study. Another study that followed up 460 rural residents for 5 years also found the proportion of metabolic syndrome to be 37.9/1000 person-years in males and 18.9/1000 person-years in females [33]. The present study included only farmers, whereas the previous study included all rural residents; it is presumed that the different findings may be attributable to 47.4% of the subjects in the previous study being unemployed. The reason why females showed a higher risk of metabolic syndrome in the present study may be attributed to several factors. First, pregnancy and childbirth have been reported to cause metabolic disorders accompanied by weight gain, increased abdominal obesity, and postpartum depression [34–36]. Since the females who participated in the present study had an average age in their 60s, the fact that most have experienced pregnancy and childbirth may have influenced the results. Second, previous studies have reported a statistically significant positive correlation between BMI and risk of metabolic syndrome [37, 38], and other studies have presented obesity as the most sensitive indicator of metabolic syndrome [39, 40]. It is presumed that females having significantly higher risk of obesity than males in in the present study may have influenced the results.

Chi-square test results for musculoskeletal pain showed that a significantly higher proportion of females had neck, hand, lower back, and leg pain compared to males. Multiple logistic regression analysis results also showed that females had a higher risk of pain than males; specifically, the odds ratios were 16.79 [95% CI, 3.09–91.30] for hand pain, and 2.34 [95% CI, 1.16–4.70] for pain in at least one body part. A previous study that investigated the risk and characteristics of musculoskeletal pain in 1013 Korean farmers found that females had a significantly higher risk of pain than males, with odds ratios of 1.77 [95% CI, 1.18–2.64] for shoulder pain, 3.88 [95% CI, 2.35–6.42] for hand pain, 2.13 [95% CI, 1.39–3.24] for lower back pain, and 1.92 [95% CI, 1.29–2.86] for leg pain [11]. The higher overall risk of pain in females shown in the previous study is similar to the present study, but the pain areas were different. This difference is postulated to be due to the present study applying NIOSH Standard 2, whereas the previous study applied NIOSH Standard 1. In a study of musculoskeletal pain in 220 Indian rice farmers, the risk of pain in females was significantly higher than that of males for shoulder, hand, lower back, and knee pain [41]. For the farmers in the present study, fruits were the main crop, while the main crop in the previous study was rice. Farming different crops is predicted to lead to differences in posture while farming, which would, in turn, lead to differences in the location of pain. In the present study, female farmers showed higher risk than males for hand pain. This may be because female Korean farmers often perform tasks that require repetitive use of the hands and fingers [27, 28]. Moreover, females in the present study showed significantly higher time spent on housework than males and, as a result, the working time, including housework, may be higher in female farmers than in male farmers. In a study that investigated the difference in musculoskeletal disorders according to gender among 358 Korean farmers, the average daily working time for female farmers (9.6 h) was longer than that of male farmers (9.2 h). Since the female farmers also tended to be solely responsible for housework, they had a greater burden [27, 28]. It is postulated that female farmers showed a higher risk of hand pain than males because housework mostly involves the use of the hands.

For psychosocial stress assessed using the PWI-SF, the chi-square tests results showed that a higher proportion of females had potential stress and high-risk stress than males. Further, females had a higher risk for high-risk stress than males (OR: 3.10 [95% CI, 1.17–8.24]). In a 2017 study that used the PWI-SF to analyze psychosocial stress factors in 3631 rural residents, females had a significantly higher risk for high-risk stress than males (OR: 2.34 [95% CI, 1.88–2.92]), which is similar to the present study [42]. In a 2011 study on 1737 rural residents, psychosocial stress was significantly higher in females than in males, which was also similar to the present study [43]. These results are postulated to reflect the characteristics associated with cultural differences regarding gender roles in Korean society and the patriarchal characteristics of Korean rural areas [44, 45]. The relatively longer working hours for female farmers are also presumed to act as a burden, resulting in increased stress [28].

For self-health awareness assessed using the SF-12, females showed significantly lower scores than males for PCS, MCS, total score, and 8 sub-items, indicating that females tended to perceive their health to be poor compared to males. Previous studies also showed similar results, where females showed lower perception of their overall self-health than males did [25, 46, 47]. Nettleton explained that performing the dual task of work and housework has a negative effect on the health of females [48]. Meanwhile, MacIntyre explained that symptoms are more readily noticed in females since they tend to be well aware of their own health, whereas males do not accept the fact that they may be ill and perceive their health to be better than it actually is [49]. In such cases, males may show relatively better scores than their actual health status, which may be the reason for the lower perception of their self-health in females than males. In other words, itis presumed that the responses to the questions might contain over- or under-estimations.

The present study has several limitations. First, the study population consisted of people from 11 rural areas in Gyeongsangbuk-do Province but, because of the small sample size from each area, it is difficult to generalize the findings for all farmers. Second, there was no investigation of the life expectancy of male and female farmers in Korea. The life expectancy of females in the general population in Korea was found to be 85.6 years in 2017, which was longer than the 79.5 years for males [2]. In a previous study conducted in the United States, the life expectancy of females in rural areas in 2005–2009 was 79.7 years, which was longer than that of males aged 74.1 years [50]. In the present study, the health status of female farmers was poorer than was that of males, but we could not confirm if they had a longer life expectancy than did males despite their poorer health status.

Despite these limitations, this study was able to compare lifestyle diseases, musculoskeletal pain, psychosocial stress, and self-health awareness to identify differences in the physical and mental health status of farmers according to gender. It also demonstrated that female farmers had higher health risks than male farmers, indicating that female farmers tend to have poorer health than male farmers. In addition, this study is significant in recognizing these differences and thus it can be used as basic data for the development of a specialized health promotion program for female farmers.

## Conclusions

While there have been many studies on the specific health issues of farmers, there have been almost no studies to date that have examined the overall difference in the health of farmers according to gender. This study was conducted to investigate the differences in health status between male and female farmers. The items that showed differences in the health status of farmers according to gender were metabolic syndrome, musculoskeletal pain, psychosocial stress, and self-health awareness. For all items that showed significant differences, female farmers showed higher risk than male farmers; thus, female farmers tended to have poorer overall health than male farmers. Therefore, when developing health promotion programs for farmers in the future, specialized programs will have to be developed to improve the health of female farmers.

## Abbreviations

ALT: Alanine aminotransferase
AST: Aspartate aminotransferase
BMI: Body mass index
BP: Bodily pain
CI: Confidence interval
FBS: Fasting blood sugar
GH: General health
HDL-C: High-density lipoprotein cholesterol
IDF: International Diabetes Federation
KOSHA: Korea Occupational Safety and Health Agency
KRW: South Korean won
LDL-C: Low-density lipoprotein cholesterol
MCS: Mental component score
MH: Mental health
NCEP ATP III: National Cholesterol Education Program’s Adult Treatment Panel III
NHIS: National Health Insurance Service
NIOSH: National Institute for Occupational Safety and Health
OR: Odds ratio
PCS: Physical component score
PF: Physical functioning
PWI-SF: Psychosocial well-being index short form
RE: Role emotional
RP: Role physical
SF: Social functioning
SF-12: 12-item short form health survey
γ-GTP: Gamma-glutamyltransferase
